# Face Painting and Enameling

**Published:** 1880-06

**Authors:** 


### FACE PAINTING AND ENAMELLING.

It is a melancholy thing to know that so many of our young ladies of the fashionable world, should be given to the habit of painting their faces at all; but especially of using dangerous poisons, and jeoparding their lives and health for the sake of producing a little higher color on their cheeks, or a little darker eyebrows. That they do it we know from what we see and hear, as well as from the advertisements in the daily newspapers. The testimony given in the recent trial of the celebrated Madame Rachel, of London, shows that she was in the habit of enamelling ladies' faces and that she was liberally paid for it!

We learn from a report, from a distinguished physician, in a recent Medical Journal, of our own country, that he had been called to the case of a young lady who was suffering from what appeared to be some disease of the spine; her physicians had treated her for such complaint, and that was understood by every one to be the nature of her illness. This physician, however, in studying her case very thoroughly, came to the conclusion, that his patient's illness was caused by the use of some poison; he named the poison which would produce the effects he saw in her case, and a searching inquiry proved that his patient had been for some time using a face-paint composed largely of this very poison! It was fortunately discovered in season to save the young lady's life.

We were some days at a fashionable hotel last winter, and sat at table directly opposite to a young lady who had had small-pox and her face was somewhat pitted. She used some composition *to fill up the pits*, and then powdered freely and seemed to think, poor thing, that no one would ever suspect the truth in her case.

Young ladies, you are injuring your health by the use of these poisonous cosmetics, and you deceive nobody. You make as great fools of yourselves as do the old men who dye their beards and hair; as far off as you can see them, you can see that their hair is colored.

